# First person – Melanie Gartz

**DOI:** 10.1242/dmm.047514

**Published:** 2020-11-13

## Abstract

First Person is a series of interviews with the first authors of a selection of papers published in Disease Models & Mechanisms, helping early-career researchers promote themselves alongside their papers. Melanie Gartz is first author on ‘Duchenne muscular dystrophy (DMD) cardiomyocyte-secreted exosomes promote the pathogenesis of DMD-associated cardiomyopathy’, published in DMM. Melanie conducted the research described in this article while a graduate student in Jennifer Strande's lab at the Medical College of Wisconsin, Cardiovascular Research Center, Milwaukee, WI, USA. She is now a postdoctoral fellow in the lab of Michael Lawlor at the Medical College of Wisconsin, Neuroscience Research Center, investigating the molecular mechanisms that underlie cardiomyopathy in Duchenne muscular dystrophy.


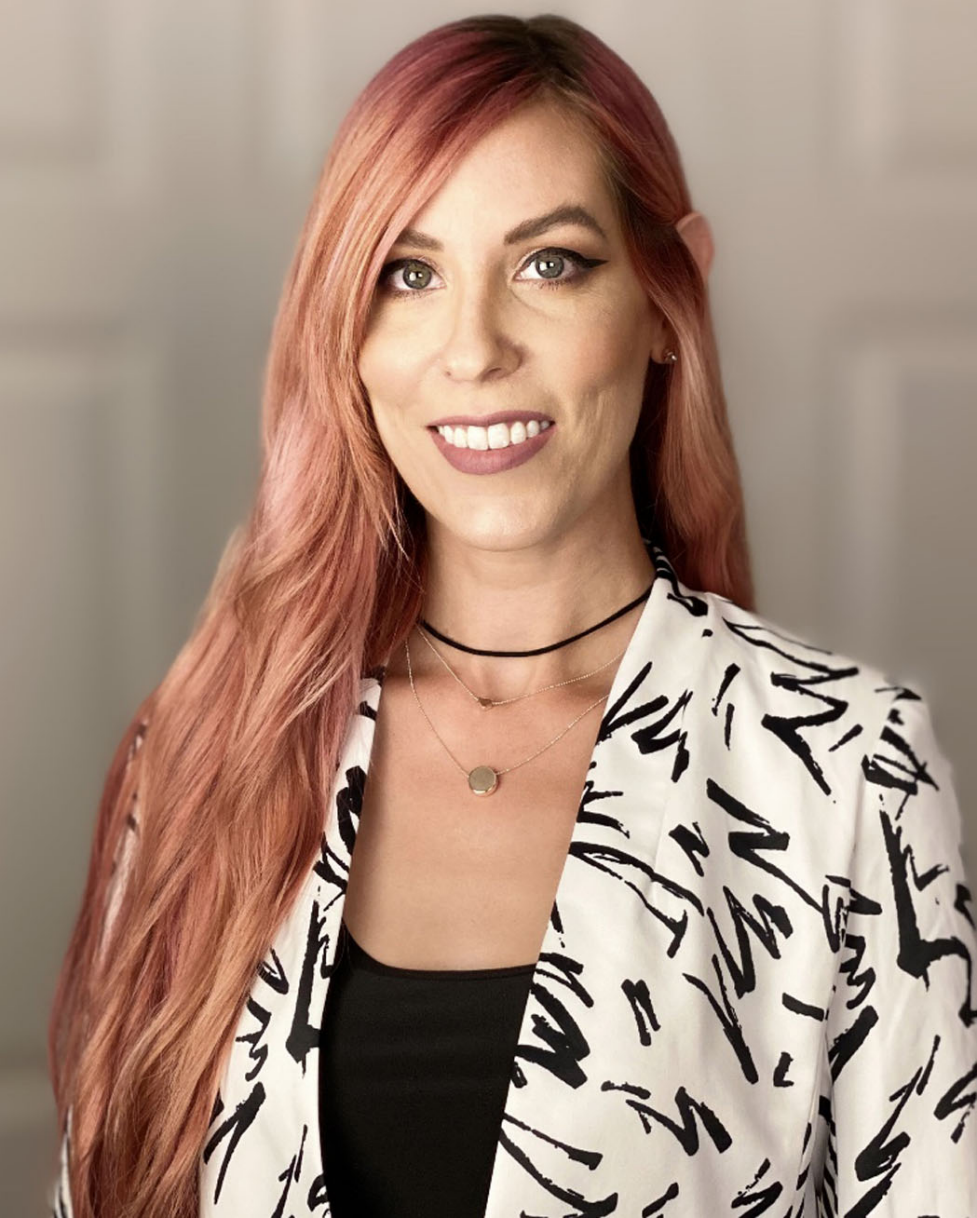


**Melanie Gartz**

**How would you explain the main findings of your paper to non-scientific family and friends?**

In this project, we wanted to explore ways by which diseased cells communicate over time and whether this communication impacted the disease environment positively or negatively. We determined that in Duchenne muscular dystrophy (DMD) cardiomyopathy, diseased cells were secreting vesicles called exosomes and the messages contained within the diseased exosomes were altered in comparison to healthy exosomes. Long-term exposure to these altered messages negatively impacted the cells' ability to respond to stressful stimuli. The findings in this study reveal a new understanding of how cells communicate during disease and identify key differences that may offer advances in therapeutic research in DMD.

**What are the potential implications of these results for your field of research?**

The findings in this study reveal differences in the effects exerted by long-term exposure to diseased versus healthy exosomes. While this study focused on DMD, there may be potential implications for a detrimental role for exosomes secreted by other diseased or injured cell types. Examining cellular communication that is occurring during disease may lead to the development of more precise therapies.

**What are the main advantages and drawbacks of the model system you have used as it relates to the disease you are investigating?**

In this project, we primarily used induced pluripotent stem cell (iPSC)-derived cardiomyocytes to model DMD cardiomyopathy. This cell-based model is ideal for interrogating disease mechanisms, as it contains a patient-specific *DMD* gene mutation. This model allows us to observe cellular consequences directly resulting from the gene mutation. Cardiac differentiations of iPSCs generally yield a >95% population of cardiomyocytes. Therefore, we can readily study our cell type of interest without confounding results due to a mixture of cell types. A limitation of this model is that it may not fully recapitulate the physiologic disease state. Therefore, we supplemented our *in vitro* studies with the *mdx* mouse, a mouse model of DMD cardiomyopathy, to examine the long-term effects of diseased exosome exposure *in vivo*.

“Currently, the most significant challenge that researchers around the world right now are facing is the COVID-19 pandemic, and how it has dramatically altered the ways in which we carry out bench research.”

**What has surprised you the most while conducting your research?**

We used three different DMD iPSC lines for experiments, and I was surprised that while all three cell lines were vulnerable to stress injury, some were more vulnerable than others. Two of the cell lines were patient derived, and one was a CRISPR gene-edited cell line. While all were dystrophin deficient as a result of the *DMD* gene mutation, they were susceptible to stress at varying capacities.

**Figure:**
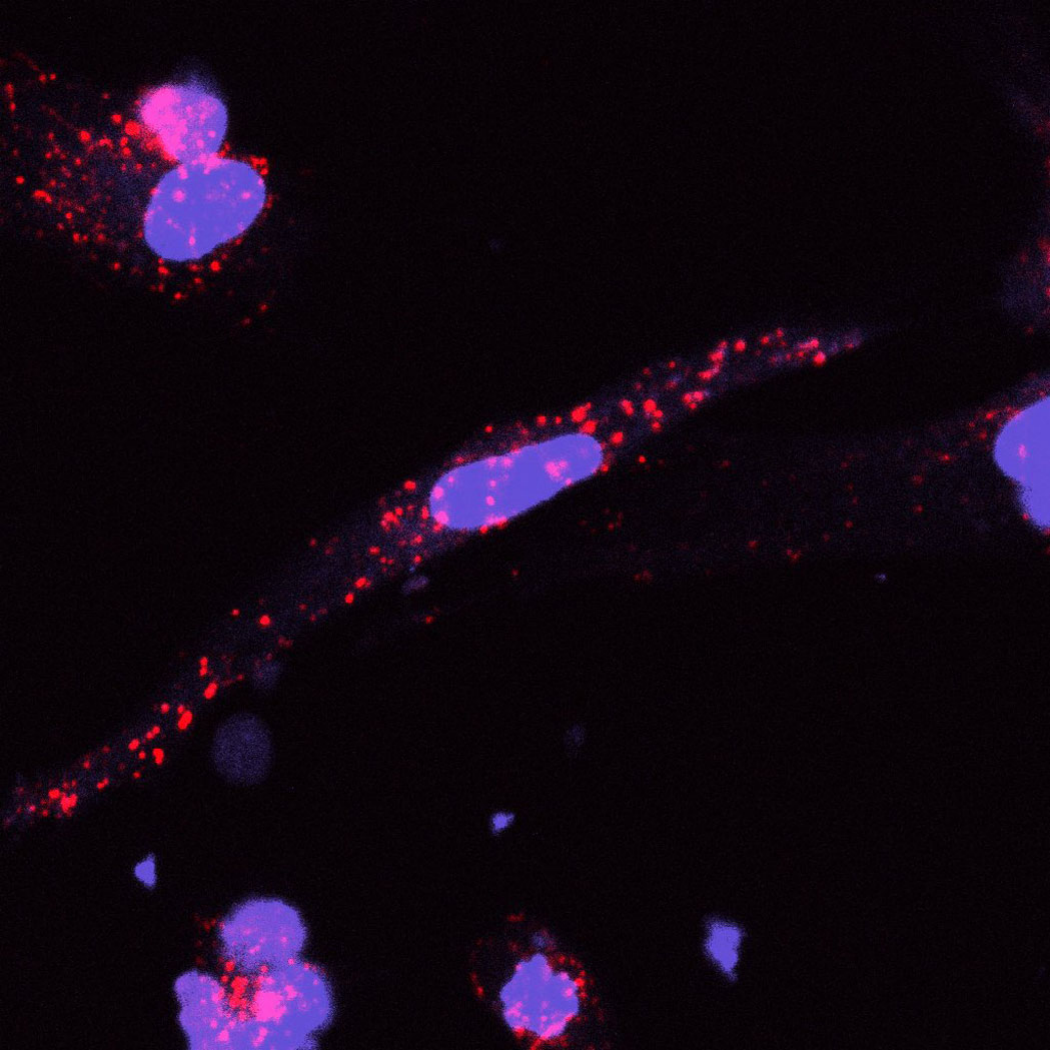
**Uptake of numerous Duchenne muscular dystrophy (DMD) exosomes (PKH26 stain, red) can be seen taken up into DMD induced pluripotent stem cell-derived cardiomyocytes (Hoechst, nuclei in blue).**

**Describe what you think is the most significant challenge impacting your research at this time and how will this be addressed over the next 10 years?**

Currently, the most significant challenge that researchers around the world right now are facing is the COVID-19 pandemic, and how it has dramatically altered the ways in which we carry out bench research. I believe that going through this incredibly challenging time as an early-career scientist will help build resilience, and lead to more unique approaches to performing scientific research in dynamic and changing situations.

**What's next for you?**

I just started my postdoctoral fellowship, and am looking forward to furthering projects to understand pathological processes in DMD. Treatments for DMD often improve the skeletal muscle pathology and function, but do little to improve cardiac function. I would like to examine skeletal and cardiac muscle more closely in DMD to potentially explain differences in treatment responsiveness.
